# PTMs as molecular encoders: reprogramming chaperones into epichaperomes for network control in disease

**DOI:** 10.1016/j.tibs.2025.07.006

**Published:** 2025-08-27

**Authors:** Feixia Chu, Sahil Sharma, Stephen D. Ginsberg, Gabriela Chiosis

**Affiliations:** 1 Department of Molecular, Cellular & Biomedical Sciences, University of New Hampshire, Durham, NH, USA; 2 Chemical Biology Program, Memorial Sloan Kettering Cancer Center, New York, NY 10065, USA; 3 Center for Dementia Research, Nathan Kline Institute, Orangeburg, NY 10962, USA; 4 Department of Psychiatry, New York University Grossman School of Medicine, New York, NY 10016, USA; 5 Department of Neuroscience & Physiology, New York University Grossman School of Medicine, New York, NY 10016, USA; 6 NYU Neuroscience Institute, New York University Grossman School of Medicine, New York, NY 10016, USA; 7 Department of Medicine, Division of Solid Tumors, Memorial Sloan Kettering Cancer Center, New York, NY 10065, USA

## Abstract

Recent discoveries reveal that post-translational modifications (PTMs) do more than regulate protein activity – they encode conformational states that transform chaperones into epichaperomes: multimeric scaffolds that rewire protein–protein interaction networks. This emerging paradigm expands the framework of chaperone biology in disease and provides a structural basis for systems-level dysfunction in disorders such as cancer and Alzheimer’s disease. This review explores how PTMs within intrinsically disordered regions drive epichaperome formation, how these scaffolds selectively regulate disease-enabling functions, and why their disruption normalizes pathological networks. By highlighting PTMs as molecular encoders of supramolecular assemblies, we propose a shift from targeting proteins to targeting network architectures that sustain and perpetuate disease – a concept with broad implications for cell biology, disease propagation, and therapeutic design.

## Beyond regulation and towards encoding

**Post-translational modifications (PTMs)** (see Glossary) have long been viewed as regulatory switches – fine-tuning protein stability, localization, or activity in response to cellular signals [1–3]. Yet mounting evidence suggests that in certain contexts, PTMs do more than modulate protein behavior: they define structural identity and reprogram molecular function [4–6]. This is particularly evident in the case of chaperone proteins, whose transformation into **epichaperomes** – stable, multimeric **scaffolding assemblies** [7,8] – relies not on mutations or overexpression, but on PTMs applied to **intrinsically disordered regions (IDRs)** [9,10].

Epichaperomes are not conventional chaperone complexes [11]. Rather than assisting in protein folding [12,13], they act as supramolecular hubs that scaffold and redirect **protein–protein interaction (PPI)** networks [8,14–17]. Their formation represents a fundamental shift in chaperone biology – from dynamic folding machines [18–21] to kinetically trapped, disease-enabling structures that persist, coordinate, and rewire functional networks at scale [8,14–17].

While chaperones are ubiquitously expressed and abundant across cells and tissues [22], epichaperomes represent a minor, disease-specific fraction of the total chaperone pool [8,14–17]. Their formation is spatially and temporally restricted, context-dependent, and reversible – making them both biologically distinct and clinically actionable [8,14–17].

Foundational evolutionary work has proposed that chaperones such as heat shock protein 90 (HSP90) buffer phenotypic variation and potentiate cellular adaptation under stress conditions [23,24]. Epichaperomes may represent a pathological co-option of this latent plasticity – stabilizing maladaptive PPI networks under chronic stress. The PTMs that drive this transformation do not merely regulate chaperone activity; they encode an alternative structural and interactional state – one that is tightly linked to disease onset, progression, and, potentially, propagation [9,10].

In this review, we synthesize recent findings that reveal how PTMs reprogram chaperones into stable, network-organizing platforms. We examine how this transformation enables large-scale, spatially constrained rewiring of PPI, and how such assemblies contribute to disease maintenance and progression. These insights offer a conceptual reframing of PTMs – from modulators of protein function to structural encoders of persistent, disease-sustaining **interactomes** – with implications for systems biology, cell fate, and therapeutic intervention.

By reframing PTMs as molecular encoders of supramolecular assemblies, this piece offers a new conceptual lens on how structural biology, network regulation, and disease pathogenesis intersect – and where PTM-defined scaffolds may be therapeutically disrupted.

## From modifications to assemblies: how PTMs drive epichaperome formation

**Figure 1.**
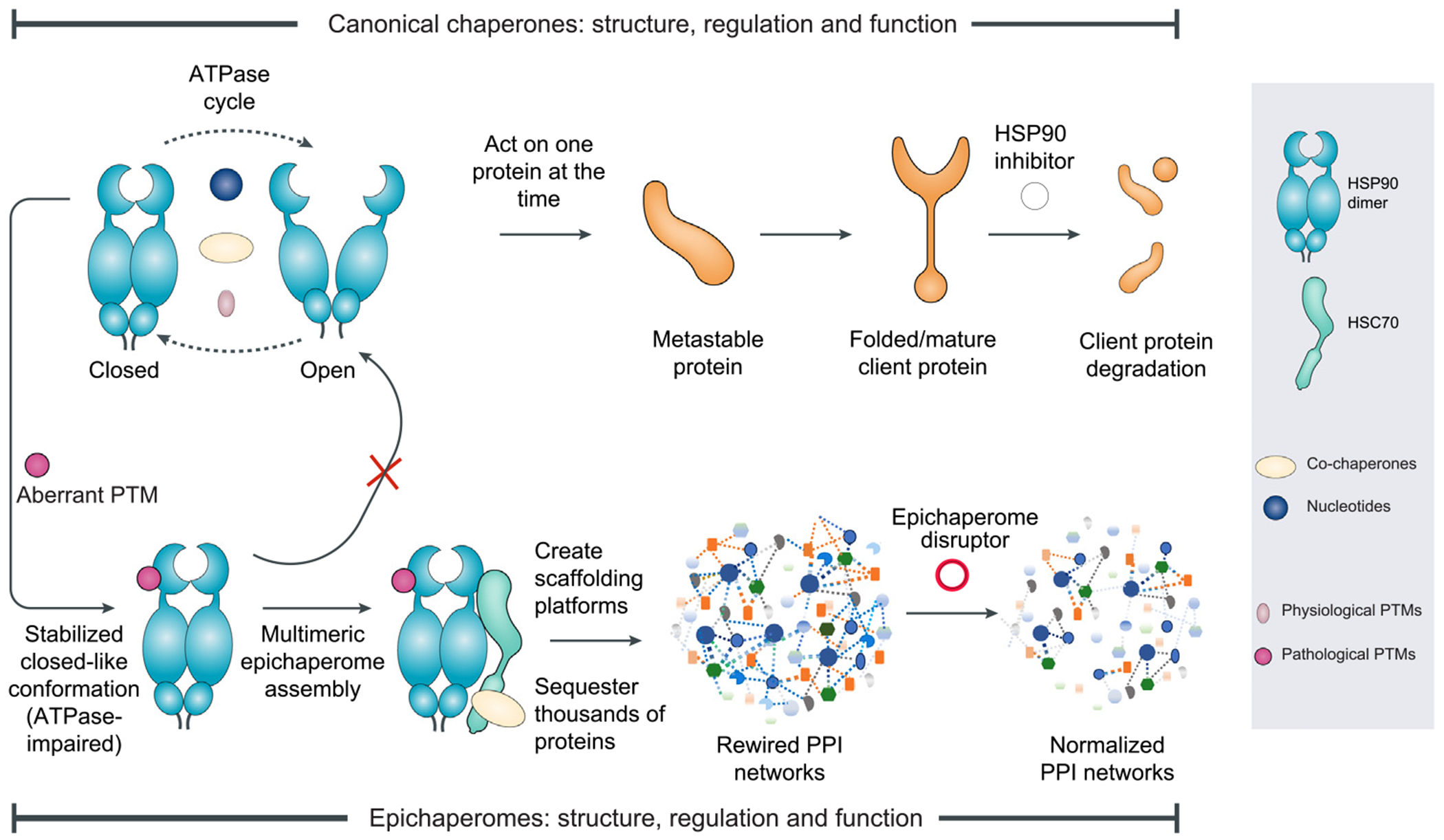
Reprogramming chaperones into epichaperomes: assembly, function, and disassembly. Canonical chaperones such as heat shock protein 90 (HSP90), heat shock cognate 70 (HSC70), and glucose-regulated protein 94 (GRP94) are dynamic, ATP-dependent machines that assist protein folding, stabilization, and degradation through transient interactions. Their activity is tightly regulated by post-translational modifications (PTMs), co-chaperones, and nucleotide cycling, allowing them to respond flexibly to cellular demands. Under physiological conditions, these chaperones act individually or in small complexes; when inhibited, their client proteins are typically degraded. In disease states, PTMs located in intrinsically disordered regions (IDRs) reprogram chaperone conformation and promote their stable incorporation into higher-order multimeric assemblies termed epichaperomes. These are not aggregates or enlarged folding complexes, but rather discrete, persistent structures that become building blocks of supramolecular scaffolding platforms. Critically, epichaperome formation disables ATPase cycling, shifting chaperones from dynamic folding machines into kinetically trapped interaction units. These epichaperome-based platforms – formed by the assembly of many epichaperomes – sequester thousands of proteins into confined, high-density compartments. This rewires PPI networks to support disease-sustaining processes such as aberrant signaling, metabolism, and synaptic dysfunction. Despite their wide impact, epichaperomes constitute a minor, PTM-defined subset of the total chaperone pool. Epichaperome disruptors like PU-H71 bind preferentially to chaperones when assembled into epichaperomes – not in their transient or canonical chaperone states. This binding kinetically traps the assembly in a non-dynamic conformation, disrupting its stability and triggering scaffold disassembly. As a result, bound proteins are released and normal protein–protein interaction (PPI) network organization is restored – a mechanism distinct from traditional chaperone inhibition.

Epichaperomes are supramolecular scaffolds formed from otherwise transient and dynamic chaperone complexes (Figure 1). Their defining feature is structural stability – not mediated by changes in protein expression or mutation, but instead through PTMs that alter the conformation and interaction behavior of key components [9–11,25,26]. Chief among these components are HSP90, heat shock cognate 70 (HSC70), and glucose-regulated protein 94 (GRP94), whose IDRs harbor key PTM sites that regulate their ability to engage in long-lived multimeric assemblies observed across diverse tissues and disease states [9–11,25,26].

Phosphorylation is one such PTM that acts on the charged-linker region of HSP90, an IDR critical for its conformational dynamics [10]. For example, phosphorylation at Ser226 and Ser255 on HSP90β – an HSP90 paralog – stabilizes a ‘closed-like’ conformation by flipping the linker into an ‘up’ position, exposing the middle domain of HSP90 and promoting stable interactions with co-chaperones such as HSC70 [10]. This conformational shift impairs HSP90’s canonical ATPase-driven folding cycle while enabling its incorporation into high-order, multimeric epichaperome structures [10]. These assemblies, in turn, sequester protein to rewire oncogenic signaling, enhancing the aggressive phenotype of tumors [8,14–17].

This interpretation is indirectly supported by earlier studies showing that phosphorylation at these same sites (Ser226/Ser255) inhibits canonical chaperone functions: phosphomimetic mutations weaken HSP90’s interaction with the aryl hydrocarbon receptor (AHR) transcription factor, reduce ligand-mediated transcriptional activity, and promote Apaf-1 release and apoptosome assembly – highlighting how these PTMs disengage client binding and predispose HSP90 to alternative, non-canonical roles [27–29]. In human embryonic kidney 293 (HEK293) cells with endogenous HSP90β knocked-out, re-introduction of the phospho-wild-type protein showed lower binding efficiency to several well-characterized HSP90 clients, whereas the non-phosphorylatable mutant (S226A/S255A) displayed stronger association with those same clients [30]. This pattern reinforces a gate-keeping role whereby Ser226/255 phosphorylation restrains classical chaperone activity and primes HSP90 for structural repurposing.

The functional consequences of this phosphoregulation were confirmed through mutational studies [10]. Cells expressing phosphomimetic HSP90 mutants (HSP90β S226E/S255E) displayed increased epichaperome formation, elevated activity of pro-survival pathways (e.g., MEK, AKT, mTOR), enhanced proliferative capacity, and mesenchymal-like features [10]. By contrast, non-phosphorylatable mutants (S226A/S255A) were deficient in these phenotypes [10], establishing a direct link between PTM status, epichaperome assembly, and pathological cellular behavior.

Notably, these phospho-switches are not only functionally required but are also selectively enriched in disease states [10,31]. Quantitative analyses of matched tumor and normal tissues normalized for HSP90 expression showed that Ser226/255 phosphorylation is elevated in tumors, when and where epichaperomes form [10]. Moreover, when the non-phosphorylatable mutant (S226A/S255A) is introduced into cells that endogenously form epichaperomes, it incorporates into the assembly and destabilizes it, acting as a dominant negative [10]. Conversely, the phosphomimetic mutant (S226E/S255E) enhances epichaperome formation, even in the presence of wild-type HSP90 [10]. These findings demonstrate that Ser226/255 phosphorylation is not merely a permissive modification, but a key determinant of epichaperome stability and cooperativity.

GRP94, another epichaperome-incorporated chaperone, is similarly regulated through PTMs [9,25,26]. Glycosylation at Asn62, a residue within its N-terminal IDR, promotes epichaperome incorporation by favoring a conformationally closed state of the ATP-lid, disrupting its normal folding cycle [26]. This stabilization supports persistent interactions with epichaperome partners [9,26,32] and drives the formation of plasma membrane-localized scaffolds [9,33,34] that may cluster oncogenic proteins, such as epidermal growth factor receptor (EGFR), to potentiate signaling [9,26]. Clustered regularly interspaced short palindromic repeats (CRISPR)-based mutagenesis of GRP94 (e.g., N62Q) confirmed that loss of glycosylation diminishes epichaperome formation and disrupts EGFR signaling platforms [9].

Together, these studies establish PTMs on IDRs as necessary and sufficient for the incorporation of HSP90 and GRP94 into epichaperomes, determining their structural roles within these assemblies and their ability to drive disease-specific functions. The flexibility of IDRs allows for multiple conformational states, and PTMs encode the transition between these states, acting as structural regulators that toggle chaperones between dynamic folding machines and stabilized scaffolding units.

This mechanism highlights a broader principle in chaperone biology, aligning with the concept of the ‘**chaperone code**’ whereby combinatorial PTM patterns govern chaperone activity, client selection, and complex formation (Box 1) [35]. In the context of disease, the PTM-driven conversion of chaperones into epichaperomes represents a potent rewiring mechanism, redirecting proteome behavior not by genetic mutation, but by dynamic conformational programming.

Thus, PTMs do not merely fine-tune chaperone activity; they can fundamentally reprogram PPI networks through the stabilization of pathological assemblies (Figure 1). These PTMs act as molecular switches, but critically, they do not merely activate or inhibit chaperone function – they encode the conditions under which chaperones abandon their canonical roles and instead engage in stable, disease-associated scaffolding.

## Mechanisms that regulate PTMs: enabling epichaperome formation

While PTMs are central to epichaperome formation, the processes that determine their spatial and temporal occurrence are regulated, not random.

In the case of GRP94, for example, N-glycosylation at silent sites such as Asn62 is typically suppressed under normal conditions but becomes selectively activated under stress [25,36,37].

Studies show that disruption or saturation of oligosaccharyltransferase activity – via endoplasmic reticulum (ER) stress, partial complex loss, or oncogenic signaling – can enable glycosylation at these otherwise silent sites [9,25,36,37]. Importantly, this PTM not only promotes but also stabilizes a pathological GRP94 conformation that favors its incorporation into epichaperomes and confers scaffolding activity not observed in its canonical form [9,26,38].

For HSP90, multiple layers of regulation converge to control its transformation into an epichaperome. A recent study identified casein kinase 2 (CK2) as a key driver of this process in cancer via phosphorylation of HSP90 at Ser226 and Ser255 (or the corresponding Ser231/Ser263 in HSP90α) [10]. Notably, inhibition of CK2 reversed HSP90 phosphorylation and reduced epichaperome assembly, underscoring its role as a disease-specific PTM regulator. CK2 is a constitutively active kinase, and its overexpression has been reported in various diseases, including cancers, infectious diseases, neurological disorders, and cardiovascular conditions [39]. Whether this CK2–epichaperome link is conserved outside cancer remains an open question.

Beyond the charged-linker sites, CK2 also phosphorylates a conserved N-domain residue (T22); this mark slows HSP90 ATPase activity, diminishes canonical folding, and sensitizes cells to HSP90 binding small molecules [40]. CK2’s reach extends beyond HSP90 itself to its co-chaperone network [39]. For example, CK2 was shown to phosphorylate the HSP90 co-chaperone folliculin-interacting protein 1 (FNIP1) on a cluster of serines, with each additional phospho-mark incrementally enhancing FNIP1–HSP90 binding and inhibiting HSP90 ATPase activity. The same stepwise phosphorylation increased the abundance and tyrosine-kinase activity of v-Src (and other clients) in proportion to the number of serines phosphorylated [41]. Together, these findings illustrate that CK2-driven PTMs at multiple points converge to decelerate the canonical folding cycle, reinforcing the view that CK2 acts as a master switch that primes the chaperone system for scaffold-like, epichaperome assemblies rather than rapid client turnover.

What regulates CK2 in this context? Earlier work revealed that MYC, a key oncogenic transcription factor, is a master regulator of epichaperome formation [8]. In inducible MYC models, toggling MYC expression in cancer cells directly altered epichaperome levels: MYC overexpression in epichaperome-low cells triggered assembly, while MYC withdrawal in epichaperome-high cells led to their disassembly [8]. Mechanistically, MYC upregulates a network of stress-adaptive programs – including transcriptional and metabolic rewiring [42] – that likely elevate CK2 activity and promote HSP90 phosphorylation. Importantly, cells with higher epichaperome levels exhibited greater sensitivity to epichaperome-targeting agents such as PU-H71, consistent with the concept that epichaperome abundance determines therapeutic vulnerability [8,11]. Thus, epichaperome formation in this setting is not driven by isolated PTMs alone, but by MYC-controlled **stress phenotypes** that induce the signaling environment – such as CK2 kinase activity – required to install the phosphorylation marks that repurpose HSP90 into a scaffolding conformation.

Transcriptional cues that drive PTM machinery and modulate chaperone and co-chaperone abundance also contribute to epichaperome formation. In T-cell acute lymphoblastic leukemia (T-ALL), Kourtis *et al.* demonstrated that the NOTCH1 oncogene transcriptionally regulates epichaperome levels [43]. Pharmacological inhibition of NOTCH1 with γ-secretase inhibitors significantly decreased epichaperome abundance and reduced sensitivity to PU-H71 [43]. This suggests that NOTCH1 regulates the formation of the epichaperome network itself, possibly through MYC-driven transcriptional programs that promote a stress-adaptive state permissive for epichaperome assembly [44]. While PTMs remain the critical determinants of structural transformation, increased expression of select co-chaperones under such programs may enhance or stabilize the multimeric assemblies required for epichaperome formation [43].

These data suggest that cellular stress not only triggers PTMs but also reconfigures the machinery responsible for their site-specific placement, enabling the conformational switch from folding chaperone to scaffolding platforms. These findings establish a causal link between oncogenic signaling, PTM machinery, and chaperone reprogramming, highlighting the epichaperome as an emergent property of the diseased cellular state. Although PTMs are necessary for epichaperome formation, they are not sufficient on their own – contextual factors such as stress-induced signaling and co-chaperone abundance ultimately govern where and when these marks are installed. Thus, upstream drivers may serve as indirect but critical regulators of epichaperome abundance and function. Importantly, this mechanism highlights a key biological advantage: regulating chaperone function through PTM-controlled assembly into supramolecular scaffolds provides a rapid, dynamic, and spatially precise means of rewiring the proteome – superior to slower, less targeted strategies such as changes in protein expression. By modifying assembly states rather than abundance, cells under stress can swiftly reorganize interaction networks to support survival, growth, or immune evasion in a context-specific manner (Box 2).

## Architecting the network: functions regulated through epichaperome assembly

Cells face a fundamental challenge: how to rapidly and reversibly reorganize their proteome in response to stress without requiring energetically costly new transcription or translation. One solution is to regulate not the expression levels of proteins but their assembly states [45–48], a strategy that epichaperomes exemplify.

As discussed previously, unlike canonical chaperones that function as transient, client-specific machines, epichaperomes form stable, supramolecular scaffolds that rewire PPIs across entire networks. But they do not do this indiscriminately. Epichaperomes emerge under chronic or intense stress – such as that associated with disease – where they selectively regulate functions that are spatially distributed, structurally complex, and highly sensitive to coordination. These include signal transduction networks [8,10,15,16], synaptic function and plasticity [14,17], mitosis, and chromatin organization [16], among others.

Intriguingly, many of these same pathways are canonically active during embryonic development, where they orchestrate cell fate, morphogenesis, and tissue patterning [49–51]. In mature cells, these programs are tightly repressed, but in disease, they are frequently reactivated [49,50]. This raises the possibility that epichaperomes do not simply emerge to manage stress, but may reflect the reawakening of a developmental scaffolding system, normally used to transiently organize the proteome during early cell states (Box 3).

Such functions share key characteristics: they operate in dense macromolecular environments, rely on rapid switching or feedback, and cannot tolerate stochastic variation. Critically, they often involve membraneless subcompartments, where diffusion and local concentration gradients – not bulk protein abundance – determine outcome [52–54].

**Figure 2.**
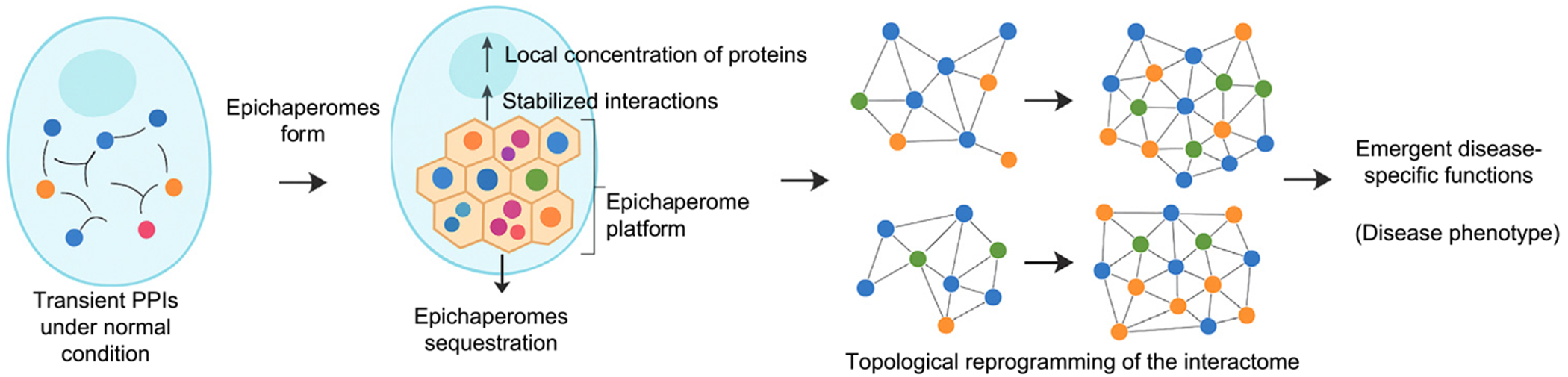
Epichaperomes reorganize the proteome by scaffolding protein–protein interaction networks to enable disease-specific functions. In healthy cells (left), protein–protein interactions (PPIs) are governed by spatial and temporal dynamics, enabling flexible, reversible coordination of cellular processes. Under chronic or pathological stress, this balance is disrupted, and chaperones assemble into epichaperomes – stable, multimeric scaffolds that sequester proteins into confined subcellular compartments (center). This sequestration increases local concentration, stabilizes otherwise transient interactions, and enforces new topological constraints on the interactome. As a result, epichaperomes reprogram network connectivity, giving rise to emergent, disease-specific functions (right). These scaffolded networks sustain pathological phenotypes such as aberrant mitosis in cancer or synaptic dysfunction in neurodegeneration. Through this mechanism, epichaperomes convert stress-induced structural changes into persistent and maladaptive network behavior.

In this context, epichaperomes may act as organizational hubs: stabilizing otherwise transient PPIs, corralling specific modules, and suppressing unwanted crosstalk [7,15,16,55]. But they also go further – by sequestering proteins into confined, high-density platforms, epichaperomes may alter the thermodynamic and spatial landscape of the cell [17]. They increase local concentrations, thus enabling low-affinity or entropically unfavorable interactions, and creating new biochemical environments in which aberrant complexes can form. This enforced proximity may rewire the topology of the interactome, giving rise to emergent, disease-specific functions that would not occur in healthy states [8,14,16,17]. This allows the pathological cell state to selectively reconfigure high-stakes pathways without invoking genome-wide reprogramming or irreversible damage (Figure 2).

Why is this advantageous to disease cells? Because it is rapid, localized, and reversible, ideal for managing stress without invoking genome-level change. Cells already maintain high basal levels of core chaperones such as HSP90 and HSC70, far exceeding the demands of routine folding [56]. This surplus is not idle – it provides a structural buffer that can be co-opted under stress, allowing chaperones to be repurposed into scaffolding assemblies without compromising proteostasis. When specific PTMs accumulate, this latent reservoir is redeployed into epichaperome structures [11,25], platforms that stabilize aberrant interactions and rewire network logic.

Cancer cells, for instance, exploit epichaperomes to enforce mitotic fidelity and stress survival signaling [8,16,57]. In neurons, epichaperomes emerge in Alzheimer’s disease and other chronic stress contexts, where their scaffolding activity disrupts synaptic organization and contributes to network dysfunction [14,17,58–60]. Rather than providing protection, these assemblies represent a maladaptive shift, stabilizing harmful interactions and blocking recovery.

This explains why epichaperomes preferentially regulate PPI networks that define and maintain the disease phenotype: they scaffold the very pathways that the pathological cell relies on for persistence [61]. Disrupting epichaperomes therefore collapses these aberrant scaffolds, releasing sequestered proteins and restoring native interaction dynamics [14,17,59,62]. As a result, PPI networks revert toward their physiological configuration, reversing the molecular underpinnings of the disease state [61].

This mode of regulation aligns with broader concepts arguing that IDRs enable stress-induced, functionally adaptive assemblies across the proteome (Box 4) [63]. Epichaperomes represent a disease-hijacked version of this principle: they take advantage of PTM-tunable IDRs to convert chaperones into structural scaffolds, exerting control not through new proteins but through new architectures.

Thus, the epichaperome is not simply a byproduct of stress – it is a stress-maladaptive pseudo-organelle or scaffolded compartment in its own right. Its emergence reflects a shift from protein-centric to network-centric control, with profound implications for how cells rewire their proteome under pathological conditions. Once formed, epichaperomes reorganize the interactome by stabilizing aberrant PPIs. This rewiring is highly selective – not random aggregation but modular editing of network topology [14,16,17]. Thus, unlike canonical HSP90/HSP70 chaperone complexes, which buffer key pathways – translation, DNA repair, and kinase signaling [56,64,65] – through fleeting, ATP-driven contacts, epichaperomes lock whole pathway modules, rewiring network topology and sustaining pathological cell states.

Data from dysfunctional Protein–Protein Interactome (dfPPI) analyses – a chemoproteomics-based systems biology platform that maps changes in PPIs by capturing epichaperomes and the proteins they sequester [66] – show that epichaperomes sequester hundreds to thousands of proteins, with pathway specificity that reflects the defining functional requirements of the cellular context or phenotype [8,14,16,17]: for example, epichaperomes scaffold networks involved in synaptic signaling and translational control in neurodegeneration, while promoting cell cycle regulation, stress adaptation, and signaling amplification in cancer [11,61,66].

PTMs appear to act upstream of this network remodeling by defining the ‘**epichaperome code**’: they could determine which chaperone components are recruited, which conformations are stabilized, and by extension, which subsets of the interactome become scaffolded. The implication is that PTMs do not just initiate epichaperome formation – they inscribe its topological output. In this view, the cell’s signaling landscape is not only responsive to PTMs but is actively restructured by them through epichaperome-mediated scaffolds.

## Therapeutic implications: targeting the PTM-encoded scaffold

Epichaperomes do not form randomly, nor do they regulate peripheral cellular processes. Instead, as discussed in the prior section, they assemble to scaffold the very functions that the diseased cell is most dependent on – those that are under high demand, structurally intricate, and coordination-sensitive. These include pathways like mitotic fidelity in cancer, or synaptic signaling in neurodegeneration, systems where even minor errors or failures in regulatory control have disproportionate consequences.

This is not coincidental. Epichaperomes selectively engage with PPI networks that are already primed for dysregulation in the disease context. Their formation reflects a structural selection process, driven by PTMs, that enables them to scaffold the exact functional modules required to maintain the pathological state [8,14–17]. Through their assembly, they stabilize key hubs and modules in these networks, locking them into a maladaptive but stable configuration. This enforced stability allows the cell to preserve its pathological state, whether it is the unchecked proliferation of a tumor cell or the persistent excitotoxic stress in a degenerating neuron [8,14–17].

Critically, this same logic explains why epichaperome disruption has disproportionately large effects [14,16,59,61]. Because epichaperomes scaffold multiple interdependent nodes within a network, their disassembly does not simply remove one interaction – it collapses entire subnetworks [14,16,59,61] (Figure 1). It breaks enforced proximity, disrupts aberrant interactions, and restores the native topological and spatial organization of the proteome. This is network normalization, achieved not by replacing proteins or reversing mutations, but by dismantling disease-sustaining scaffolds.

In this light, the function of epichaperomes is not to manage stress, but to reprogram the cell’s interaction space in a way that hardwires its disease phenotype. Their disruption thus restores the potential for physiological plasticity, a reset of the cellular network logic, not its composition [14,16,59,61].

The structural specificity of epichaperomes conferred by PTMs offers both a therapeutic opportunity for resetting these networks and a unique therapeutic window. It is now well established that chaperones such as HSP90, GRP94, and HSC70 form the core of epichaperome assemblies in multiple diseases, including Alzheimer’s disease [14,17], Parkinson’s disease [58,62], traumatic brain injury [60], and various cancers [8,10,15,16,57,67,68]. These insights have guided the development of small molecules capable of selectively disrupting these disease-specific assemblies. Unlike traditional inhibitors, which generally target enzymatic activity or folding function, **epichaperome disruptors** disassemble the stable scaffolding platforms that define these pathological structures [11].

Zelavespib (also known as PU-H71), the first-in-class epichaperome disruptor, illustrates this mechanism [7,11]. It binds to HSP90 preferentially when the chaperone is incorporated into an epichaperome, kinetically trapping the assembly and initiating its disassembly [69]. In doing so, PU-H71 restores normal PPIs without altering overall expression levels or substantially interfering with physiological chaperone function [10,14,57,62]. While PU-H71 binds the ATP pocket of HSP90, its binding and biological activity are determined by the context of that pocket. CK2-driven phosphorylation at Ser226/255 shifts HSP90 into a closed, epichaperome-competent conformation [10]; this severely reduces binding of classical inhibitors such as luminespib (also known as NVP-AUY922), as well as ATP itself [70]. PU-H71, by contrast, shows increased affinity for this phosphorylated form [10]. Thus CK2-driven phosphorylation both promotes epichaperome formation and sets a selectivity filter.

Building on PU-H71, a growing portfolio of epichaperome-targeting agents is now in development. PU-AD (icapamespib), which also targets HSP90-containing epichaperomes [11,71], moved to Phase 2 clinical trials for Alzheimer’s disease and Phase 1 trials for glioblastoma [72]. PU-H71 (zelavespib) has progressed through early-stage trials for various cancers, showing activity in metastatic breast cancer and relapsed or refractory acute myeloid leukemia [67,68]. Other disruptors, including LSI-137 (targeting epichaperomes via HSC70) [16] and PU-WS13 (targeting epichaperomes via GRP94) [26,38], are advancing in preclinical studies. These compounds are characterized by their ability to preferentially engage epichaperomes at disease sites for extended durations, while rapidly dissociating from normal, transient chaperone assemblies. This **kinetic selectivity** underlies both their therapeutic potential and favorable safety profiles [11].

This selective vulnerability – assembly-dependent drug action – suggests that PTMs define not only the formation of pathological scaffolds, but also their druggability. As the field moves toward therapies that address network dysfunction rather than single targets, PTM-encoded epichaperomes stand out as precision hubs for intervention.

## Concluding remarks

The epichaperome is more than a stable chaperone complex – it emerges as a structurally encoded platform, assembled and maintained by a pattern of PTMs that rewrite the cell’s interaction logic. These PTMs convert dynamic chaperones into scaffolding platforms, rerouting PPI networks, coordinating stress responses, and, potentially, spreading pathology across tissue. In this light, PTMs may be viewed as a supramolecular language – one that encodes structural and functional cellular states through conformational programming. Understanding this language that governs epichaperome formation and function may unlock new frameworks for decoding disease and restoring homeostatic network states.

While much remains to be explored, several open questions emerge about the potential for epichaperomes to coordinate dysfunction across space, not only within a cell but potentially across connected systems (and even across time; see Outstanding questions).

Epichaperome scaffolds have traditionally been studied for their intracellular effects – stabilizing aberrant PPIs, rerouting functional networks, and amplifying stress signals within a given cell.

However, growing evidence suggests they may also participate in broader circuit-level dysfunction [14,17]. In mouse models, epichaperome formation has been shown to follow a defined spatiotemporal trajectory: emerging in select brain regions early in disease and progressively extending as pathology advances [14,17].

A provocative question arises: can epichaperome assemblies, once formed, propagate dysfunction to adjacent or distant cells, circuits, or tissues? Unlike prions, epichaperomes are not aggregates – they are structured, multimeric scaffolds – but they may share a key feature: the ability to encode and transmit a pathological state. Given their stability and selective cargo retention, it is plausible that epichaperomes direct the trafficking of disease-enabling material – mitochondria, signaling proteins, extracellular vesicles, and RNA-binding complexes – to propagate dysfunction. If true, this represents a new model of non-genetic information transfer: a PTM-defined supramolecular code carried across cells.

Understanding the extent to which epichaperomes influence not just single-cell function but multicellular networks will be essential to fully define their role in disease. If their PTM-defined architecture governs both intracellular remodeling and intercellular signaling potential, epichaperomes may represent a new class of scaffolds with the capacity to encode and extend dysfunction beyond their site of origin. If true, this would not only position epichaperomes as vehicles of dysfunction but elevate PTMs as the foundational language that enables their encoding and transmission.

## Box 1. PTMs as modulators of chaperone conformation and function

Chaperones are highly responsive to post-translational modifications (PTMs), which modulate their conformational states, interactions, and functional outputs across stress and signaling contexts. Recent reviews and several research articles highlight how phosphorylation, acetylation, small ubiquitin-like modifier (SUMO) conjugation (SUMOylation), and other PTMs act as a ‘chaperone code’ – a regulatory language used by cells to tune chaperone activity, complex formation, and substrate specificity and can alter conformational dynamics and biological outcomes in a context-dependent manner [7,35,64,73–76].

While these works focus on canonical chaperone complexes and their role in proteostasis, they establish a clear precedent: chaperones are highly susceptible to PTM-mediated remodeling, and cells exploit this to reconfigure chaperone assemblies and their downstream effects. How these regulatory mechanisms intersect with or enable epichaperome formation remains to be clarified, but the susceptibility of chaperones to PTM-driven structural reprogramming is now well established.

Why are chaperones especially vulnerable to PTM-based regulation? One reason lies in their intrinsic conformational plasticity [18–20]. Chaperones must cycle through defined conformational states to carry out their functions – binding, remodeling, and releasing substrates. These transitions are exquisitely sensitive to local chemical environment and structural inputs. PTMs, especially when located in IDRs, may act as minimal and reversible triggers that shift the energetic landscape of these conformations. This makes chaperones highly tunable – but also susceptible – to PTM-driven reprogramming. In disease, cells may co-opt this vulnerability to shift chaperones into alternate assemblies, such as epichaperomes, that serve new structural roles unrelated to folding. This PTM sensitivity may thus represent both a feature of normal chaperone biology and a liability exploited in pathology.

## Box 2. Regulating assembly, not abundance: a structural strategy for proteome rewiring under stress

While the traditional view has emphasized that cells respond to stress by altering transcriptional programs or increasing protein expression, mounting evidence suggests an additional regulatory layer: modulation of protein assembly states. Rather than synthesizing new proteins, cells can repurpose existing ones into multimeric scaffolds that support new functions. This assembly-based regulation enables rapid, localized adaptation to stress and disease with minimal biosynthetic cost.

### From expression to configuration

Traditional models of proteostasis have emphasized protein abundance as the primary mode of regulation: increase chaperones under heat shock; boost antioxidants under oxidative stress [77,78]. However, structural biology and systems-level interactomics increasingly show that changing how proteins interact – their oligomerization, conformational state, or network embedding – can more powerfully influence function [7,61,79–83]. This shift in understanding is particularly relevant in disease contexts, where PTMs and protein crowding under stress create conditions ripe for new assemblies to form.

### A structural analogy

If increasing protein expression is like stockpiling bricks, modulating assembly is like reorganizing those same bricks into a fortress, shelter, or bridge, depending on the need. One does not require new components – only new blueprints and interactions. This analogy captures the advantage of regulatory plasticity without biosynthetic cost.

### The epichaperome as a model of assembly-based regulation

Epichaperomes exemplify this principle. They are not new proteins but structural reorganizations of existing chaperones like HSP90 and GRP94, stabilized by disease-associated PTMs. Their formation reflects a broader stress-induced rewiring strategy: to deploy existing proteins into multimeric scaffolds that stabilize aberrant protein–protein interactions and sustain pathological signaling.

### Support from structural dynamics theory

This perspective aligns with the work of Ruth Nussinov, Monika Fuxreiter, and others who have championed the view that protein function arises from dynamic conformational ensembles [84–86]. Rather than existing in a single native state, proteins sample multiple conformations, and PTMs or partner interactions can shift this equilibrium – stabilizing specific functional or dysfunctional states without changing expression levels. Fuxreiter’s studies of fuzzy complexes [85] and Nussinov’s work on allostery and conformational selection [86] both support the idea that misassembly, not misexpression, is a common root of pathology. In this framework, disease can emerge when normally transient or low-population states become kinetically trapped, forming aberrant structures such as epichaperomes.

### Functional advantages

Assembly-state regulation offers key benefits over expression-level control:

- Speed: structural shifts occur faster than transcription or translation.
- Specificity: assemblies form in defined cellular locales or stress contexts.
- Reversibility: assemblies can disassemble once the stress resolves.
- Efficiency: function is changed without the energetic cost of new synthesis.

### Implications

In stress biology, and especially in disease conditions, the ability to rewire proteome function through conformational shifts and supramolecular assembly is a rapid and structurally encoded mechanism to reorganize the proteome under duress, enabling cells to shift functional states without requiring de novo synthesis. Epichaperomes represent one such mechanism, where existing proteins are structurally transformed into scaffolds that stabilize aberrant protein interactions. Therapeutically, this highlights the value of targeting disease-specific assemblies rather than individual proteins, offering precision without disrupting homeostasis.

Thus, under conditions of stress, regulating the assembly state of proteins provides a structurally encoded, efficient, and adaptive route to proteome remodeling – one that may be more advantageous than changes in gene expression alone.

## Box 3. Epichaperomes as misappropriated developmental scaffolds

Epichaperomes are typically viewed as stress-induced scaffolds that rewire protein networks in disease. However, their presence in pluripotent stem cells (PSCs) [10,62] – and absence in most adult cells [8,14,57,71] – suggests a deeper origin: epichaperomes may be developmentally encoded assemblies that organize protein–protein interaction (PPI) networks during embryogenesis to coordinate rapid and large-scale changes in cell state (Figure I).

In PSCs, epichaperomes regulate proliferation and plasticity, at least in part through control of OCT4 [10,62]. Notably, their formation is absent in most normal, non-pluripotent cells – including adult neurons and other cell types with high functional demand [14,17,71] – indicating that epichaperome assembly is not a general stress or workload response, but a state-specific mechanism tied to cellular plasticity and identity transitions. Supporting this, a subset of the pathways regulated by epichaperomes in diseases such as cancer and Alzheimer’s – including Wnt, Notch, MYC, and chromatin remodeling networks [8,14,16,17] – are also highly active during development [42,49,50].

Moreover, conditions that induce epichaperomes in disease (e.g., MYC, NOTCH1, CK2 activation) [8,10,43] mimic molecular features of PSCs [10], indicating that diseased cells partially reacquire molecular features of developmental states – that is, revert to a developmental-like program [49–51].

In this view, epichaperomes are not inherently pathological, but become maladaptive when their developmental logic is hijacked and locked into place by post-translational modifications (PTMs). This persistence stabilizes disease-associated PPI networks, disrupting proteome flexibility and enforcing pathological identity.

Understanding epichaperomes as misappropriated developmental assemblies offers a new lens through which to view network-level dysfunction and reveals opportunities to restore normal PPI network organization by dismantling these persistent, disease-specific scaffolds.

## Box 4. IDRs as tunable scaffolds: the convergence of PTMs and network control

Intrinsically disordered regions (IDRs) are increasingly recognized as molecular scaffolds – not through stable 3D structure, but through their conformational plasticity and regulatory flexibility [63,87]. These properties make IDRs ideal substrates for post-translational modifications (PTMs) that dynamically modulate protein interactions, localization, and assembly. In the context of epichaperomes, IDRs act as structural switches that enable disease-associated scaffolding.

- Structural plasticity: IDRs exist as ensembles of conformations that can be tuned by cellular conditions. In chaperones such as HSP90 and GRP94, IDRs like the charged linker or N-terminal tails are highly flexible regions that help regulate interaction behavior [88,89]. PTMs within these IDRs shift conformational equilibria, enabling stable integration into multimeric epichaperome assemblies [10,26].
- PTM-rich regulation: IDRs are hotspots for PTMs such as phosphorylation and glycosylation. These modifications act as molecular encoders – controlling whether a chaperone adopts a dynamic, folding-competent role or a scaffolded, interaction-networking role within an epichaperome. This repurposing is governed not by substrate engagement but by PTM-induced structural stabilization.
- Scaffold assembly and compartmentalization: many IDR-containing proteins undergo phase separation to create biomolecular condensates [90]. While epichaperomes do not phase-separate *per se*, their assembly mimics certain features: spatial sequestration, increased local concentration of interactors, and emergent compartment-like functions that regulate signaling and stress responses.

Taken together, IDRs provide the molecular grammar for stress-adaptive assembly. Epichaperomes hijack this grammar – via PTMs – to lock dynamic chaperones into persistent scaffolds that enforce pathological protein–protein interactions. Understanding how IDRs are repurposed in this way may reveal therapeutic opportunities to selectively dismantle disease-enabling architectures without impairing physiological chaperone function.

## Highlights

Post-translational modifications (PTMs) can convert molecular chaperones into epichaperomes – stable scaffolds that restructure protein–protein interaction (PPI) networks in disease.

PTMs on chaperone intrinsically disordered regions act as molecular switches, reprogramming their conformation and interactions to drive network-level dysfunction.

Epichaperomes enable large-scale PPI rewiring without altering protein levels or sequence, emerging in cell states like pluripotency and disease.

Epichaperomes are stable, ordered assemblies that scaffold high-demand pathways central to disease, including mitosis, metabolism, and synaptic signaling.

This reframes disease as a disorder of PPI networks and supports a network-centric strategy for therapeutic intervention.

## Outstanding questions

What specific PTM patterns define the transition of chaperones into epichaperomes across different cell types and disease states?

How do PTMs control the spatial organization, stability, and topological output of epichaperomes in distinct subcellular compartments?

Can epichaperomes influence other cells through vesicular transport or organelle transfer, and if so, do PTMs regulate their transmissibility?

Is there a PTM-encoded signature that determines the selectivity of protein sequestration into epichaperomes?

How reversible are epichaperome-mediated network changes once scaffolded, and does reversal require full disassembly of the scaffold?

## Glossary

Chaperone code: a conceptual framework describing how combinations of post-translational modifications (PTMs) regulate the structure, activity, and interactions of molecular chaperones, determining their conformational states and interaction profiles.
Epichaperome code: the specific pattern of PTMs on epichaperome components (often on IDRs) that determines scaffold composition, stability, and interaction topology. Analogous to the chaperone code but functionally specialized for pathological scaffolding.
Epichaperome disruptors: small molecules that selectively bind epichaperome-incorporated chaperones to kinetically trap and disassemble the scaffold without interfering with normal chaperone function. Examples include PU-H71, PU-AD, and PU-WS13.
Epichaperomes: disease-specific, multimeric scaffolds formed from otherwise transient chaperone complexes (e.g., HSP90, HSC70, GRP94) that are stabilized through PTMs and reorganize protein–protein interaction (PPI) networks.
Interactome: the full set of PPIs within a cell. Epichaperomes restructure the interactome by stabilizing aberrant PPIs and rewiring network connectivity under stress conditions.
Intrinsically disordered regions (IDRs): protein segments that lack a fixed three-dimensional structure. IDRs often serve as regulatory hubs for PTMs and facilitate conformational plasticity and multivalent interactions.
Kinetic selectivity: a drug-targeting principle where compounds differentiate between molecular targets not by binding site alone, but by differences in binding/unbinding kinetics – such as prolonged engagement with epichaperome-bound HSP90 versus transient chaperone forms.
Post-translational modifications (PTMs): covalent chemical modifications to proteins (e.g., phosphorylation, glycosylation) that regulate protein structure, stability, localization, and interaction potential. In epichaperome biology, PTMs act as structural switches that drive assembly into scaffolds.
Protein–protein interactions (PPIs): physical contacts between proteins that govern most cellular processes. In disease, epichaperomes can reprogram PPIs by sequestering proteins into scaffolded environments that stabilize non-physiological interactions, alter interaction preferences, and reorganize network topology.
Scaffold/scaffolding assembly (biological): a structural platform that organizes and stabilizes multiple proteins or complexes, enabling spatial and temporal control of cellular functions. Epichaperomes act as disease-specific scaffolds that reorganize cellular networks.
Stress phenotype: a cellular state induced by external or internal stressors (e.g., oncogene activation, proteotoxic stress) characterized by altered signaling, metabolism, and protein interaction landscapes.
